# Mapping the neural patterns of verbal repetition: an activation likelihood estimation meta-analysis

**DOI:** 10.1007/s00429-026-03119-3

**Published:** 2026-05-30

**Authors:** Ariane Awana, Marcelo L. Berthier, María José Torres-Prioris, Diana López-Barroso

**Affiliations:** 1 Cognitive Neuroscience and Aphasia Unit, University of Malaga, 29010 Malaga, Spain; 2 Department of Psychobiology and Methodology of Behavioural Sciences, Faculty of Psychology and Speech Therapy, University of Malaga, 29010 Malaga, Spain; 3 IBIMA-Plataforma BIONAND, 29010 Málaga, Spain; 4 Language Neuroscience Research Laboratory, Faculty of Psychology and Speech Therapy, University of Málaga, 29010 Malaga, Spain

**Keywords:** Language, Verbal repetition, Word repetition, Pseudoword repetition, Neuroimaging, ALE meta-analysis

## Abstract

Verbal repetition involves transforming heard speech into articulatory motor output and constitutes a core language function integrating receptive and expressive processes within tight temporal constraints. This integrative nature underlies its importance for language acquisition and learning and accounts for its diagnostic relevance in acquired and developmental language disorders. Despite extensive investigation, the consistent neural architecture supporting verbal repetition has remained insufficiently established due to methodological heterogeneity across studies. Here, we conducted a coordinate-based meta-analysis using Activation Likelihood Estimation (ALE) on 27 neuroimaging experiments (380 participants; 440 activation foci) to identify brain regions consistently engaged during verbal repetition in healthy adults, to compare word and pseudoword repetition, and to assess hemispheric lateralization. Across all conditions, verbal repetition consistently engaged a robust, bilaterally distributed fronto-temporo-parietal network encompassing auditory-phonological regions of the superior temporal cortex, sensorimotor cortices of the precentral and postcentral gyri, and medial frontal motor regions including the SMA and the preSMA. Lateralization analyses revealed a predominant left-hemispheric contribution across conditions, with a stronger leftward bias for pseudoword than for word repetition, despite largely bilateral engagement. Direct contrasts indicated stimulus-dependent modulation within this partially shared dorsal network: pseudoword repetition showed greater involvement of auditory associative, premotor, and subcortical regions, whereas word repetition preferentially engaged primary auditory and medial frontal motor regions. Together, these findings provide a comprehensive overview of the neural systems supporting verbal repetition, highlighting a shared dorsal auditory-motor network that is flexibly modulated as a function of stimulus familiarity.

**Supplementary Information:**

The online version contains supplementary material available at 10.1007/s00429-026-03119-3.

## Introduction

Verbal repetition involves the reproduction of heard speech, encompassing single words, sequences of words, and unfamiliar phonological material. Although it may appear to be a straightforward function, it relies on complex cognitive and neural processes that map auditory information into motor representations necessary for articulation (Hope et al. 2014). At the cognitive level, this involves acoustic and phonological analysis, the temporary maintenance of phonological representations, and their mapping onto the motor system for speech production (Majerus 2013; Hope et al. 2014). Beyond its fundamental role in speech, verbal repetition is also crucial for language acquisition, as it supports the learning of new words (Gathercole 2006; López-Barroso et al. 2013, 2015; López-Barroso and De Diego-Balaguer 2017; Orpella et al. 2022) and contributes to phonological short-term memory (Jacquemot and Scott 2006; Majerus 2013). In addition to its role in typical language processing, verbal repetition also has significant clinical relevance. It is central to the clinical classification of aphasic syndromes (Albert et al. 1981), has prognostic value (Hosomi et al. 2009), and guides rehabilitation strategies (Schlaug et al. 2009). Alterations of repetition are also a hallmark of language disorders. Repetition may be pathologically excessive or uncontrolled, a behavior known as echolalia, which is commonly observed in individuals with post-stroke aphasia (Berthier et al. 2018; Torres-Prioris et al. 2019; López-Barroso et al. 2023) and primary progressive aphasia (Ota et al. 2021). Conversely, selective impairments of repetition are a defining feature of conduction aphasia, in which patients exhibit disproportionate difficulty repeating spoken material despite relatively preserved comprehension and fluent speech (Albert et al. 1981). Taken together, the cognitive, developmental, and clinical dimensions of verbal repetition underscore the importance of delineating the neural systems that are consistently engaged during this function.

The dual-stream model of language processing provides a useful framework for understanding verbal repetition. According to this model, speech and language processing occurs along two main pathways: the dorsal and the ventral streams. The *dorsal stream* supports auditory-motor integration, which is crucial for verbal repetition and speech production (Behroozmand et al. 2015), while the *ventral stream* is involved in lexico-semantic mapping. This functional and anatomical distinction has led to the theoretical prediction that word repetition may rely more heavily on ventral stream processes, whereas pseudoword repetition should preferentially engage dorsal auditory-motor mechanisms (Saur et al. 2008; Majerus 2013; Moritz-Gasser and Duffau 2013). However, this dissociation is most clearly expected when word repetition is achieved via lexical-semantic access followed by speech production, rather than through a purely auditory-phonological transformation. In fact, empirical findings remain mixed: some studies report graded differences in activation within overlapping regions during word and pseudoword processing (Mechelli et al. 2003; Raettig and Kotz 2008; Hartwigsen et al. 2013), whereas others point to distinct activation patterns for each stimulus type (Yoo et al. 2012; Palomar-García et al. 2017).

Beyond stimulus-level contrasts, the issue of hemispheric specialization is also critical. While the left hemisphere has long been considered dominant for language (Knecht et al. 2000a, b; Vikingstad et al. 2000), the dual-stream model makes more nuanced predictions about hemispheric specialization. Specifically, it posits a left-lateralized dorsal auditory-motor integration circuit and a bilaterally organized ventral lexico-semantic stream, findings further supported by neuroimaging-based studies (Saur et al. 2008; Assaneo et al. 2019). Verbal repetition, which may require the interaction of both streams, therefore offers a critical test case for examining hemispheric asymmetries. Although right-hemisphere involvement has frequently been reported in patients with left-hemisphere damage, its role in recovery remains highly debated, with some studies suggesting a compensatory contribution (Berthier et al. 2012; Forkel et al. 2014) and others considering it maladaptive (Heiss and Thiel 2006; Postman-Caucheteux et al. 2010). In contrast, its contribution to verbal repetition in the intact brain remains insufficiently understood.

Despite numerous neuroimaging studies investigating verbal repetition, findings remain heterogeneous, and no synthesis has yet determined which brain regions are consistently engaged across tasks. This study aims to fill this gap by identifying the brain regions consistently activated in functional studies of verbal repetition in neurotypical adults. This gap is particularly relevant given the clinical importance of repetition: clarifying its neural bases can guide intraoperative monitoring to prevent post-surgical aphasia, identify reliable targets for non-invasive stimulation therapies, and inform repetition-based rehabilitation approaches that are widely used in aphasia treatment. Additionally, given its strong link to short-term and working memory (Gathercole et al. 1992; Jacquemot and Scott 2006; Lopez-Barroso et al. 2011; Majerus 2013), repetition training helps address short-term memory deficits that impact language function (Salis et al. 2015, 2017). Improvements in verbal short-term memory have been shown to correlate with better outcomes across multiple language domains (Harris et al. 2014; Eom and Sung 2016), further reinforcing the clinical and cognitive significance of repetition.

### The present study

The present study addresses these issues by identifying brain regions that are consistently involved in verbal repetition, both as a general function and across different experimental conditions in neurotypical adults. As an integrated linguistic function, verbal repetition engages a widespread brain network. This complexity, combined with the essential interconnectivity of brain regions required for successful repetition, underscores the need to investigate it as a unified process (Moritz-Gasser 2021). To this end, we conducted a systematic literature search and performed a coordinate-based meta-analysis of functional neuroimaging studies of verbal repetition (single words or pseudowords, word lists, meaningful sentences, or Jabberwocky sentences). By synthesizing the existing body of research, we aimed to draw consolidated conclusions and minimize the influence of individual study biases (Haidich 2010). The meta-analysis employed an Activation Likelihood Estimation (ALE) approach (Eickhoff et al. 2009). The analysis was guided by three objectives. First, to assess repetition as a general function, including repetition tasks of different types of stimuli. Second, to differentiate between the repetition of stimuli with semantic content (i.e., words) and those without semantic content (i.e., pseudowords). Finally, to address the ongoing debate on the role of the right hemisphere in language, we extracted lateralization indices (LIs) to investigate the differential contributions of the right and left hemispheres to the repetition process.

## Materials and methods

### Literature search

**Fig. 1:**
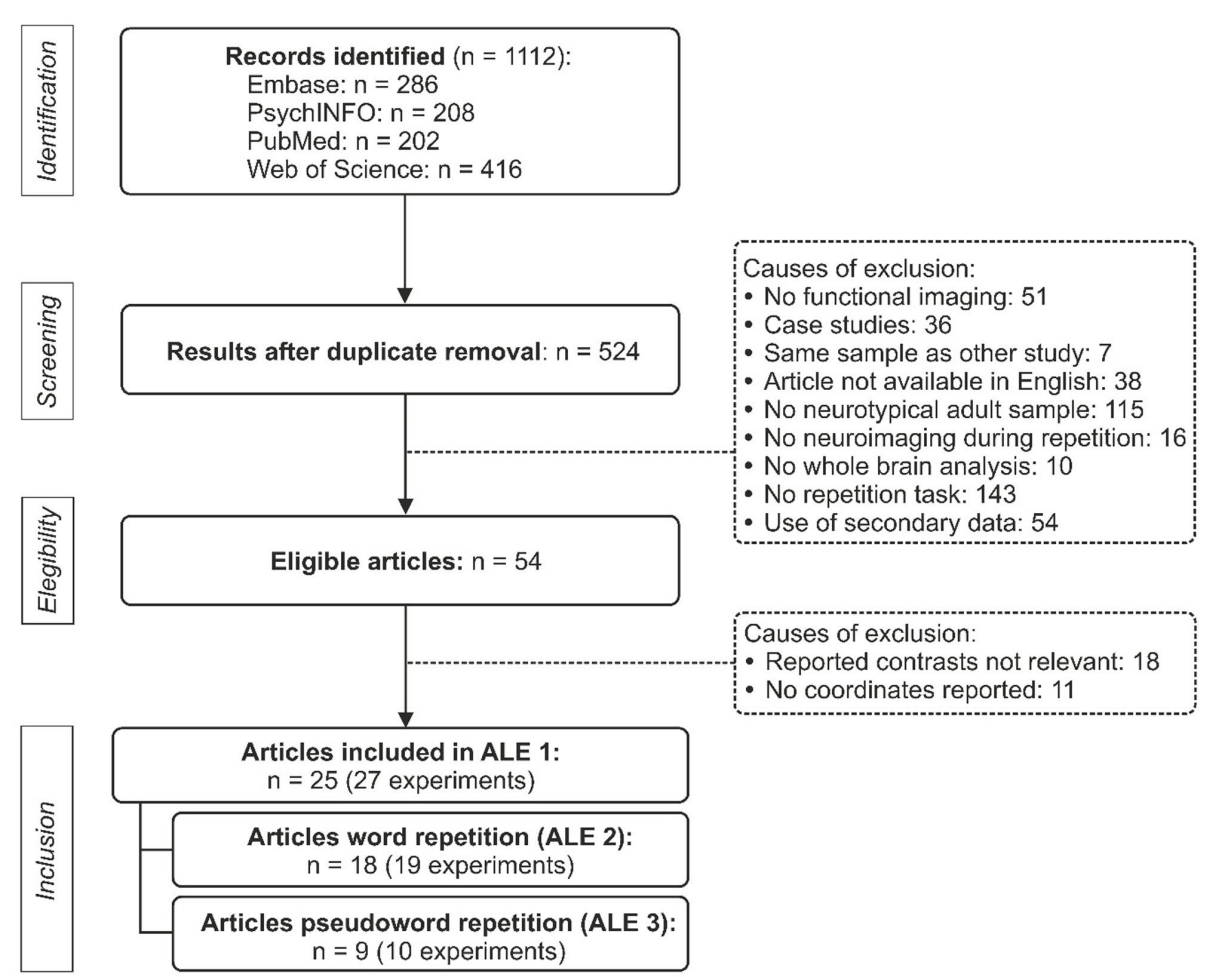
PRISMA flowchart of the ALE meta-analysis’s study literature search. *ALE* Activation Likelihood Estimation

Searches for relevant articles were conducted using the following databases: PubMed, PsycInfo, Web of Science, and Embase. These databases were chosen based on research into the optimal database combination for literature searches in systematic reviews (Bramer et al. 2017; Gusenbauer and Haddaway 2020). The search terms used to identify articles reporting functional neuroimaging studies during a verbal repetition task included: (“speech repetition” OR “sensory-motor integration” OR “audio-motor integration” OR “verbal repetition” OR “word repetition” OR “pseudoword repetition” OR “pseudo-word repetition” OR “non-word repetition” OR “nonword repetition” OR “phrase repetition” OR “sentence repetition” OR “syllable repetition”) AND (fMRI OR neuroimaging OR “functional neuroimaging” OR “functional magnetic resonance imaging” OR PET OR “positron emission tomography”). Only articles written in English were considered. Searches were conducted up to May 27th, 2025, yielding 1112 records identified across all databases (see PRISMA flowchart in Fig. 1). After removing duplicates both automatically and manually, the search identified 524 articles. Data management was performed using the systematic review web application RAYYAN (Ouzzani et al. 2016).

### Inclusion criteria

The results were initially screened based on their titles and abstracts, followed by a thorough review of the full-text articles to ensure they met the following inclusion criteria: (1) The sample was composed of more than one healthy adult participant. Studies with children, teenagers, neurologically impaired, or clinical populations were excluded; (2) The study used functional neuroimaging during a verbal repetition task: functional magnetic resonance imaging (fMRI) or positron emission tomography (PET) was performed; (3) The article was written in English; (4) Whole-brain analysis was conducted for neuroimaging data. Studies that only performed region of interest (ROI) analyses were excluded; (5) Results were reported using standard stereotactic coordinates (Montreal Neurological Institute [MNI] or Talairach space); (6) Contrasts reported should reflect functional brain processing during verbal repetition, relative to a baseline condition. Verbal repetition conditions included overt or covert repetition of words in the participants’ native or non-native language, pseudowords/nonwords (i.e., a sequence of at least two syllables without associated meaning), or sentences. Baseline conditions included rest or control tasks such as listening or producing a fixed control utterance (e.g., saying one specific word) in response to stimuli. Studies directly contrasting two repetition conditions were excluded, as these reflect differences between repetition types rather than neural activity specifically related to the repetition process.

This screening resulted in 54 eligible articles that employed a verbal repetition task while performing neuroimaging via fMRI or PET. The study selection flowchart and the specific reasons for exclusion during screening are shown in Fig. 1. If any article did not report the MNI or Talairach coordinates of the contrast of interest, the corresponding author was contacted to request the missing data. After this step, 25 articles were included in the analysis of general repetition (ALE 1, Table 1).

**Table 1:** Studies included in the ALE meta-analyses

Exp	Article	Imaging method	Mean age (range/SD)	Sample (women) ^†^	Hand	Lang	Contrast	Foci	ALE
1	Abo et al. (2004)	fMRI	21.33 (20–22/0.82)	6 (0)	R	Japanese	Word repetition (overt) > Rest	20	ALE 1, ALE 2
2	Becker et al. (1994)	PET	nr (25–40/nr)	4 (nr)	R	nr	Word repetition (overt; 3 items) > Rest	15	ALE 1. ALE 2
3	Burton et al. (2001)	fMRI	30 (22–49/nr)	10 (8)	9R, 1 L	English	Word repetition (overt) > Reverse speech listening + saying “crime”	4	ALE 1, ALE 2
4	Cowell et al. (2000)	PET	48 (27–67/nr)	12 (6)	R	English	Number repetition (overt) > Rest	6	ALE 1, ALE 2
5	Hervais-Adelman et al. (2015)	fMRI	25 (18–33/nr)	50 (26)	43R, 7 L	French/English	Sentence shadowing^§^ (overt) > Passive listening	31	ALE 1, ALE 2
6a	Hope et al. (2014)	fMRI	31.4 (20–45/nr)	25 (12)	R	English	Word repetition (overt) > Rest	58^**‡**^	ALE 1, ALE 2
6b	Hope et al. (2014)	fMRI	31.4 (20–45/nr)	25 (12)	R	English	Pseudoword repetition (overt) > Rest	43^**‡**^	ALE 1, ALE 3
7	Howard et al. (1992)*	PET	nr (18–70/nr)	12 (5)	R	English	Word repetition (overt) > Reverse speech listening + saying “crime”	9	ALE 1, ALE 2
8	Isenberg et al. (2012)	fMRI	23 (18–30/nr)	17 (7)	R	English	Pseudoword repetition (covert) > Pseudoword listening	30	ALE 1, ALE 3
9	Kenyon et al. (2024)	fMRI	45.4 (18–65/15.3)	14 (nr)	R	English	Word repetition (overt) > Rest	16^**‡**^	ALE 1, ALE 2
10	Klein et al. (2006)	PET	22 (nr/nr)	10 (5)	R	English/French	Word repetition (overt; first and second language) > Rest	16^**‡**^	ALE 1, ALE 2
11	Liégeois et al. (2003)	fMRI	25.3 (20–28/nr)	5 (2)	4R, 1 L	English	Word repetition (overt) > Rest	1	ALE 1, ALE 2
12	Liégeois et al. (2011)	fMRI	nr (nr/nr)	4 (2)	nr	English	Nonword repetition (overt) > Listening to noise	14	ALE 1, ALE 3
13	Marchina et al. (2018)	fMRI	52 (30–69/nr)	12 (5)	R	English	Word/phrase repetition (overt) > Rest	10	ALE 1, ALE 2
14	McGettigan et al. (2011)	fMRI	25 (19–36/nr)	17 (9)	nr	English	Pseudoword repetition (overt) > Humming tones	6	ALE 1, ALE 3
15	Ohyama et al. (1996)	PET	58.3 (nr/8.1)	6 (1)	R	Japanese	Word repetition (overt) > Rest	13	ALE 1, ALE 2
16	Okada and Hickok (2009)	fMRI	nr (18–44/nr)	23 (nr)	R	English	*Jabberwocky* sentence repetition (covert) > Passive *Jabberwocky* listening	13	ALE 1, ALE 3
17	Price et al. (1996)	PET	nr (22–33/nr)	6 (nr)	R	English	Word repetition (overt) (at 20 and 40 w.p.m) > Rest	24^**‡**^	ALE 1, ALE 2
18	Price et al. (1996)	PET	nr (28–62/nr)	4 (nr)	R	English	Word repetition (overt) > Listening to words + saying “crime”	9	ALE 1, ALE 2
19	Rosso et al.( 2014)	fMRI	61 (20–75/nr)	24 (nr)	R	French	Word repetition (overt) > Rest	8	ALE 1, ALE 2
20	Shuster et al. (2014)	fMRI	22.8(nr/2.4)	11 (nr)	R	English	Pseudoword repetition (overt) > Listening to noise	9	ALE 1, ALE 3
21	Shuster et al. (2014)	fMRI	56.5(nr/7.1)	12 (7)	11R, 1 L	English	Pseudoword repetition (overt) > Listening to noise	3	ALE 1, ALE 3
22	Szenkovits et al. (2012)	fMRI	24.8(nr/5.5)	20 (12)	R	English	Pseudoword repetition (overt) > Saying “yes” to buzzes	25	ALE 1, ALE 3
23	Tremblay & Small (2011)	fMRI	25 (20–29/nr)	21 (11)	R	English	Sentence repetition (overt) > Sentence listening	20	ALE 1, ALE 2
24	Weiller et al. (1995)	PET	35(27–50/nr)	6 (0)	R	German	Pseudoword repetition (covert) > Rest	4	ALE 1, ALE 3
25	Wikman et al. (2022)	fMRI	25.6 (19–39/nr)	17 (9)	R	Finnish	Sentence shadowing^§^ (overt) > Rest	9	ALE 1, ALE 2
26	Wiseman et al. (1999)	PET	40.7 (nr/7.6)	10 (0)	nr	English	Word repetition (overt; 1 and 3 items) > Fixation	3	ALE 1, ALE 2
27a	Yoo et al. (2012)	fMRI	22.8 (18–34/nr)	22 (11)	R	Korean	Word repetition (overt) > Rest	11^**‡**^	ALE 1, ALE 2
27b	Yoo et al. (2012)	fMRI	22.8 (18–34/nr)	22 (11)	R	Korean	Pseudoword repetition (overt) > Rest	10^**‡**^	ALE 1, ALE 3

*ALE *Activation Likelihood Estimation, *fMRI *Functional magnetic resonance imaging, *PET *Positron emission tomography, *R *Right, *L *left, *nr* not reported, *Exp* Experiment,* w.p.m* Words per minute, *Hand* Handedness, *Lang* Language, *SD* Standard deviation

^*^Peak coordinates were taken from a reanalysis of data reported in Price et al. (1996)

^†^Sample size refers to the effective number of participants included in the analysis from which coordinates were extracted

^**‡**^For articles reporting more than one contrast of interest with the same subjects, foci were pooled into one experiment (for all: 9, 10, 17; for ALE 1: 6a, 6b, 27a, 27b)

^§^Sentence shadowing refers to immediate overt repetition of spoken sentences during ongoing auditory input

###  Meta-analytic strategy and grouping

Three ALE meta-analyses were performed. The first analysis was the most comprehensive one and included all the experiments (ALE 1: general repetition), pooling together different forms of verbal repetition (single words, single pseudowords, sentences and Jabberwocky sentences). Based on prior empirical findings (Hanley et al. 2004; Yoo et al. 2012; Palomar-García et al. 2017) and predictions from contemporary language models (e.g. Hickok and Poeppel 2007), the subprocesses underlying word and pseudoword repetition may differ. Therefore, independent analyses for word and pseudoword repetition were conducted, as subsets of the general repetition dataset. These analyses examine potential spatial dissociations suggested in prior research, with the understanding that ALE is designed to detect consistent spatial convergence of reported peaks across studies rather than differences in activation magnitude. Accordingly, ALE 2 focused exclusively on contrasts involving stimuli with semantic content (word repetition), whereas ALE 3 focused on contrasts involving stimuli without semantic content. Although distinctions between pseudowords and nonwords may exist on their phonological resemblance to real words, the current literature does not yet provide a sufficient number of task-controlled experiments to reliably dissociate these stimulus types in a coordinate-based meta-analysis. Therefore, pseudowords and nonwords were grouped into a single category (referred to throughout the manuscript as *pseudoword repetition*) to capture neural convergence associated with repetition of non-semantic speech material. To mitigate concerns about potential instability due to the number of available studies, we evaluated robustness across all three ALE analyses (ALE 1–3) using jackknife sensitivity analyses.

###  Analyses

####  Activation Likelihood Estimation (ALE) analysis

The ALE technique for quantitative coordinate-based meta-analyses of functional neuroimaging results was applied to investigate spatial convergence across experiments. The ALE algorithm assesses the overlap between activation foci reported in functional neuroimaging studies by modelling each focus as a three-dimensional Gaussian probability distribution centered on its reported coordinates. For each experiment, these distributions are combined to generate a modeled activation map, which reflects the spatial uncertainty associated with that experiment’s reported activations. ALE scores are then computed by combining modeled activation maps across experiments, allowing identification of brain regions where convergence exceeds that expected by chance under a random-effects framework (Eickhoff et al. 2009, 2012). The resulting ALE maps represent spatially convergent activation patterns across experiments.

The three analyses (ALE 1, ALE 2, ALE 3) were performed with the BrainMap GingerALE software Version 3.0.2 (https://www.brainmap.org/index.html; Eickhoff et al. 2009; Turkeltaub et al. 2012). Since peak coordinates entered in the ALE meta-analysis must be expressed in a common stereotactic space (Talairach or MNI), coordinates originally reported in Talairach space were converted into MNI space using the *icbm2tal* transformation function implemented in GingerALE. Then, for the creation of convergence activation maps in ALE 1, ALE 2 and ALE 3, we applied an uncorrected voxel-wise cluster-forming threshold of *p* < 0.001, combined with a cluster-wise family-wise-error (FWE) corrected threshold of *p* < 0.05 based on 1000 random permutations. This thresholding approach provides an optimal balance between sensitivity and specificity in ALE analyses (Eickhoff et al. 2016). We used the MNI152 template with a less restrictive mask for computation. Peak coordinates of the significant ALE clusters were anatomically labeled using the Julich-Brain probabilistic cytoarchitectonic atlas v3.1 (Amunts et al. 2020), accessed via EBRAINS Atlas Viewer, and are reported in Tables 2, 3, 4, 5 and 6, allowing assignment to fine-grained cortical areas beyond macroanatomical gyri. For each peak, the region with the highest probability of overlap was reported. Cluster-level anatomical characterizations based on the Talairach Daemon atlas (as provided by GingerALE), including the percentage of each lobe and gyrus encompassed by each cluster, are reported in the Supplementary Material.

####  Contrast analysis

For the contrast analysis between words and pseudowords, an uncorrected p value of 0.05 with 10,000 permutations and a minimum volume of 100 mm^3^ were defined as threshold parameters. Given that the input files for the contrast analysis were already conservatively thresholded, a less stringent threshold was chosen for this additional examination, following the approach used in previous studies (Papitto et al. 2020). Since the goal of the contrast analysis was to test for differences in converging foci between word and pseudoword repetition, subtraction analyses for word repetition > pseudoword repetition and pseudoword repetition > word Repetition were conducted. These contrasts were considered exploratory, given that the number of experiments contributing to the pseudoword condition was below the minimum recommended (Eickhoff et al. 2016).

####  Evaluation of robustness: Jackknife sensitivity analysis

The robustness of the results of ALE 1, ALE 2 and ALE 3 was further explored to avoid biases related to studies with small samples, results driven by outliers, or selective reporting of positive results. To this end, we adopted a jackknife sensitivity analysis or leave-one-out strategy (Quenouille 1956), commonly used in meta-analyses of neuroimaging data (Radua and Mataix-Cols 2009; Müller et al. 2018; Enge et al. 2020). Thus, *n* different ALE meta-analyses were run, excluding one experiment at the time in each iteration (i.e., 27 trials with 26 experiments for ALE 1; 19 trials with 18 experiments for ALE 2; and 10 trials with 9 experiments for ALE 3). Results were then compared with the original ALE in terms of appearance, size, and parameters of significant voxel clusters. Through this process it can be investigated whether specific studies disproportionately drive the outcome, thereby reducing the stability of the findings. This procedure was applied equally to ALE 1, ALE 2 and ALE 3, providing an additional safeguard to ensure that even subgroup analyses (ALE 2 and ALE 3) reflected stable and reproducible patterns.

####  Lateralization indices

To explore functional lateralization in verbal repetition, weighted LIs were computed for all three ALE maps. The LIs were calculated using AveLI (Matsuo et al. 2012), a validated method that has been shown to be resistant to outliers and noise. This index uses the value of each voxel within bilateral region-of-interest (ROI) masks as a threshold to compute subordinate LIs (sub-LI) according to the following formula: sub-LI = (Lt - Rt)/(Lt + Rt), with Lt (left) and Rt (right) referring to the sums of the ALE values above threshold in the ROIs. The average of all sub-LIs is then computed as the AveLI: AveLI = Σ(sub-LI)/VN, with VN being the total number of voxels with positive values within both ROIs (Matsuo et al. 2012). The AveLI analysis was computed for the output from ALE 1, ALE 2 and ALE 3 at the whole brain level by defining two anatomical masks corresponding to the right and left hemispheres. The masks were created using the WFU_Pickatlas tool Version 3.0.5 for SPM12 (https://www.nitrc.org/projects/wfu_pickatlas/; Maldjian et al. 2003) that uses Talairach Daemon database atlases as a base (Lancaster et al. 2000). LI values below − 0.2 were considered evidence of right lateralization; LI values above 0.2 were considered evidence of leftward lateralization; and LI values between − 0.2 and 0.2 were considered to show a bilateral pattern (Seghier 2008).

## Results

###  Included Studies

From the initial 1112 articles identified in the first step of the systematic review, 25 articles were included (yielding 27 independent experiments). Previous research indicates that to achieve consistent effects (i.e., effects present in roughly one-third of the underlying population) in coordinate-based meta-analyses using ALE, a minimum of 17 experiments should be included (Eickhoff et al. 2016). Accordingly, the overall analysis pooling all repetition conditions (ALE 1) meet methodological recommendations. A detailed flowchart of the systematic review process, including exclusion criteria, is provided in Fig. 1.

The selected articles were published between 1992 and 2024 and enrolled a total of 380 participants. Of these, 10 subjects were reported as left-handed (2.63%). All experiments together provided 440 foci. In the studies that reported age (*n* = 21), the mean was 34.33 years (SD = 13.59). On average, 15.17 foci were reported (SD = 12.68). Two articles (i.e., Price et al. 1996; Shuster et al. 2014) reported two independent experiments each, bringing the total number of experiments included in ALE 1 to 27 (9 PET and 18 fMRI; Fig. 1; Table 1). In cases where more than one repetition contrast was reported for the same subjects (Price et al. 1996; Klein et al. 2006; Yoo et al. 2012; Hope et al. 2014; Kenyon et al. 2024), peak coordinates were pooled into a single experiment, following the procedure described by Turkeltaub et al. (2012). Whenever available, the repetition > rest contrast was selected, otherwise, the contrast that best reflected the aim of the meta-analysis was chosen (Müller et al. 2018) (Table 1).

ALE 2 included 19 experiments from 18 articles, and therefore also meet methodological recommendations regarding the minimum number of contributing experiments. ALE 3 included 10 experiments from 9 articles (Fig. 1 and Table 1). Two articles (Yoo et al. 2012; Hope et al. 2014) contributed to both ALE 2 and ALE 3 since they reported relevant contrasts for both pseudoword and word repetition. The coordinates derived from those contrasts were pooled together and counted as one experiment for ALE 1. Given the limited number of experiments contributing to the pseudoword condition (*n* = 10), results from ALE 3 should be interpreted as preliminary (Eickhoff et al. 2016).

###  Activation likelihood estimation results

####  ALE 1: general repetition results

**Fig. 2:**
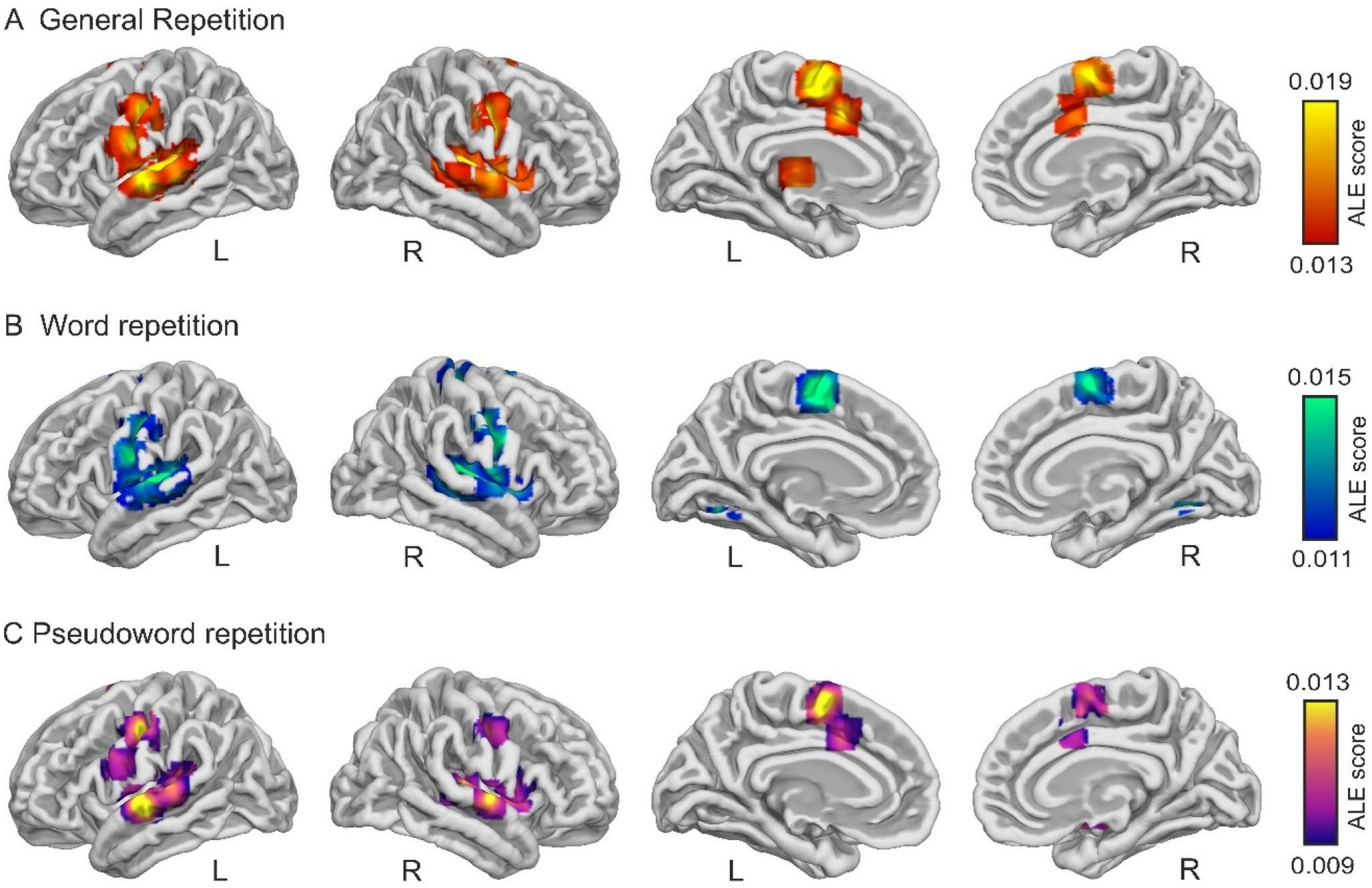
Significant activation clusters derived from the ALE meta-analysis of functional neuroimaging studies of verbal repetition: **A** General repetition (ALE 1), **B** Word repetition (ALE 2), and **C** Pseudoword repetition (ALE 3). Results are shown at *p* < 0.05 (cluster-level FWE corrected). *L* Left, *R* Right, *ALE* Activation Likelihood Estimation

In the general repetition analysis pooling all repetition conditions, seven clusters showed significant convergence for verbal repetition (cluster-level FWE-corrected at *p* < 0.05; Fig. 2A; Table 2 and Table S1). Cytoarchitectonic peak labeling revealed that cluster 1 encompassed a posterior cytoarchitectonic subdivision of the primary motor cortex (4b), within the precentral gyrus (PreCG), extending into secondary auditory cortex of the STG (Te 2.1 and Te 2.2), as well as primary somatosensory cortex (3b) in the postcentral gyrus (PostCG), the parietal operculum (Op4), and the inferior parietal lobule (PFcm; overlapping posterior supramarginal gyrus). Cluster 2 was located in the right temporal cortex, involving secondary auditory regions, including posterior and med-anterior portions of the STG (Te 2.1 and Te 2.2), and the superior temporal sulcus (STS1). Cluster 3 was centered in the medial frontal cortex, corresponding to the supplementary motor area proper (6mp; SMA). Cluster 4 involved right sensorimotor regions, primarily encompassing the primary somatosensory cortex in the PostCG (3b) with extension into the PreCG (4a). Cluster 5 was located in the medial frontal cortex within the anterior cingulate cortex (ACC), whereas cluster 6 was located in the right frontal operculum (Op6). Finally, cluster 7 corresponded to a left subcortical cluster involving the anterior intralaminar nuclei of the thalamus (CL), with additional involvement of the reticular nucleus (Rt).

**Table 2:** Results for ALE 1: general repetition

Cluster	Size(mm^3^)	ALE peak	Z peak	x	y	z	Peak label	Contributing experiments
1	15120	0.039	7.01	−48	−12	36	L PreCG (4p)	1, 2, 3, 4, 5, 6, 8, 9, 10,12, 13, 14, 16, 17, 19, 20,21, 22, 23, 24, 25, 26, 27
		0.034	6.35	−56	−18	6	L STG (Te 2.1)	
		0.034	6.33	−56	−26	6	L STG (Te 2.2)	
		0.026	5.24	−48	−30	8	L STG (Te 2.2)	
		0.024	4.91	−58	−4	22	L PostCG (3b)	
		0.019	4.15	−56	−6	10	L POperc (Op4)	
		0.013	3.21	−54	−42	26	L IPL (PFcm)	
2	6536	0.032	6.05	48	−24	8	R STG (Te 2.2)	1, 2, 4, 5, 6, 10, 13, 14,17, 19, 22, 23, 24, 25, 27
		0.028	5.60	48	−32	8	R STG, post. division*	
		0.024	5.04	62	−8	0	R STG (Te 2.1)	
		0.018	4.03	62	−28	2	R STS (STS1)	
3	3232	0.045	7.77	−6	−4	58	L SMA (6mp)	1, 3, 5, 6, 16, 17, 18, 20, 22, 23, 25, 27
4	2712	0.029	5.69	52	−8	26	R PostCG (3b)	4, 5, 6, 9, 13, 20, 22, 23
		0.027	5.45	48	−10	38	R PostCG (3b)	
		0.018	4.07	54	−4	42	R PreCG (4a)	
5	1464	0.024	5.02	−6	10	40	L CG (ant. division)*	5, 6, 8, 16, 18, 20, 22
6	1288	0.023	4.88	48	6	0	R frontal operculum (Op6)	1, 2, 5, 6, 8, 16
7	1056	0.024	5.04	−12	−18	4	L thalamus, ant. intralaminar nuclei (CL)	5, 6, 8, 10, 17, 22
		0.014	3.40	−20	−14	8	L thalamus, reticular nucleus (Rt)	

Peak coordinates are reported in MNI space. Cytoarchitectonic labels were assigned using the Julich-Brain probabilistic atlas (v3.1)

*L* Left, *R* Right, *PreCG* Precentral gyrus, *STG* Superior temporal gyrus, *STS* Superior temporal sulcus, *PostCG* Postcentral gyrus, *POperc* Parietal operculum, *IPL* Inferior parietal lobule, *SMA* Supplementary motor area, *CG* Cingulate gyrus

*For peak coordinates falling within cytoarchitectonic transition zones (GapMaps) and for which no probabilistic assignment to a specific cortical area was available, anatomical labeling was performed using the Harvard-Oxford cortical atlas, a macroscopic atlas based on gyral and sulcal anatomy, as available in MRIcron

####  ALE 2: word repetition results

Nine significant clusters were identified for word repetition (cluster-level FWE-corrected *p* < 0.05; Fig. 2B; Table 3 and Table S2). Cluster 1, the largest cluster, was located in the left temporal cortex and encompassed secondary auditory regions of the STG (Te 2.1 and Te 2.2), with additional involvement of primary auditory cortex in Heschl’s gyrus (Te 1.1 and Te 1.2). Cluster 2 was located in the right temporal cortex and involved secondary auditory regions of the STG (Te 2.2), extending into the parietal operculum (Op4). Cluster 3 involved left sensorimotor regions, encompassing primary motor cortex in the PreCG (4p) with extension into primary somatosensory cortex in the PostCG (3b). Cluster 4 was centered in the medial frontal cortex and involved the SMA proper (6mp) and preSMA (6ma), with bilateral involvement but a relative predominance in the left hemisphere. Cluster 5 involved right sensorimotor regions, primarily encompassing primary somatosensory cortex in the PostCG (3b). Cluster 6 involved primary somatosensory (3b) and primary motor (4a) cortices at a more dorsal location. Cluster 7 and 8 were confined to the cerebellum, with cluster 7 located in the left cerebellar hemisphere and cluster 8 in the right cerebellar hemisphere. Finally, cluster 9 was located in the right insular cortex (Id6).

**Table 3:** Results for ALE 2: word repetition

Cluster	Size(mm^3^)	ALE peak	Z peak	x	y	z	Peak label	Contributing experiments
1	6024	0.034	6.79	−56	−18	6	L STG (Te 2.1)	1, 2, 3, 4, 5, 6a, 10, 13,15, 17, 19, 25, 26, 27a
		0.032	6.56	−56	−26	6	L STG (Te 2.2)	
		0.016	3.99	−52	−4	2	L Heschl (Te 1.2)	
		0.015	3.96	−40	−32	12	L Heschl (Te 1.1)	
2	5816	0.028	6.06	50	−24	6	R STG (Te 2.2)	1, 2, 4, 5, 6a, 10, 15, 17,19, 23, 25, 27a
		0.024	5.45	52	−18	2	R STG (Te 2.2)	
		0.020	4.83	58	−6	4	R POperc (Op4)	
		0.012	3.42	46	−8	8	R POperc (Op4)	
3	3792	0.028	5.95	−46	−12	34	L PreCG (4p)	4, 5, 6a, 9, 10, 13, 17, 19,23, 25
		0.021	4.87	−58	−4	22	L PostCG (3b)	
4	2632	0.034	6.91	−6	−4	60	L SMA (6mp)	1, 3, 5, 6a, 17, 18, 23, 25,27a
		0.021	4.88	4	−2	62	R preSMA (6ma)	
5	1904	0.025	5.59	54	−8	26	R PostCG (3b)	4, 5, 6a, 9, 13, 23
		0.023	5.29	48	−10	38	R PostCG (3b)	
6	848	0.020	4.83	20	−28	62	R PostCG (3b)	5, 6a, 9, 23
		0.014	3.77	22	−26	72	R PreCG (4a)	
7	824	0.023	5.19	−14	−62	−20	L cerebellum	5, 6a, 10, 19
		0.011	3.17	−24	−62	−22	L cerebellum	
8	784	0.025	5.54	16	−62	−18	R cerebellum	5, 6a, 9, 10
9	736	0.017	4.30	44	8	0	R insula (Id6)	1, 2, 5, 6a, 10

Peak coordinates are reported in MNI space. Cytoarchitectonic labels were assigned using the Julich-Brain probabilistic atlas (v3.1)

*L* Left, *R* Right, *STG* Superior temporal gyrus, *POperc* Parietal operculus, *PreCG* Precentral gyrus, *PostCG* Postcentral gyrus, *SMA* Supplementary motor area, *preSMA* Pre-supplementary motor area

####  ALE 3: pseudoword repetition results

Twelve significant clusters showed convergent activation during pseudoword repetition (cluster-level FWE-corrected *p* < 0.05; Fig. 2C; Table 4 and Table S3). The largest cluster (cluster 1) was located in the right putamen. Cluster 2 was located in the left posterior superior temporal region, corresponding to the superior temporal sulcus (STS1), at the interface between the STG and the middle temporal gyrus. Cluster 3 involved left primary somatosensory cortex in the PostCG (3b). Cluster 4 covered the left inferior parietal lobule (PFcm), with extension into secondary auditory cortex of the STG (Te 2.2). Cluster 5 was located in the left medial frontal cortex and involved the preSMA (6ma). Cluster 6 was located in the temporal cortex and its main peak was centered at the temporo-parietal junction, involving the interface between the STG and supramarginal gyrus. Cluster 7 was centered in the left PreCG (6v3), with a secondary probabilistic assignment to the IFG, consistent with cytoarchitectonic uncertainty at the PreCG-IFG boundary. Cluster 8 was located in the right STG and corresponded to secondary posterior auditory cortex (Te 2.1). Cluster 9 involved the right frontal operculum (Op6). Cluster 10 was located in the right temporal cortex and involved primary auditory cortex in the Heschl’s gyrus (Te 1.1), with additional involvement of the superior temporal sulcus (STS1). Cluster 11 was located in the left medial frontal cortex within the ACC, and cluster 12 involved right primary somatosensory cortex in the PostCG (3b).

**Table 4:** Results for ALE 3: pseudoword repetition

Cluster	Size(mm^3^)	ALE peak	Z peak	x	y	z	Peak Label	Contributing experiments
1	1760	0.017	4.69	28	−4	−8	R putamen*	6b, 8, 22
		0.017	4.68	26	−2	−4	R putamen*	
		0.015	4.23	20	8	4	R putamen*	
2	1696	0.023	5.74	−60	−12	−2	L STS (STS1)	6b, 12, 14, 16, 22, 27b
3	1360	0.020	5.21	−52	−12	42	L PostCG (3b)	6b, 14, 16, 20, 22
4	1304	0.015	4.17	−52	−34	14	L IPL (PFcm)	6b, 8, 14, 16, 24
		0.013	3.81	−44	−32	12	L STG (Te 2.2)	
5	1269	0.019	4.93	−4	0	60	L preSMA (6ma)	6b, 16, 21, 22, 27b
		0.018	4.89	−4	−2	56	L preSMA (6ma)	
6	1112	0.021	5.39	−62	−28	6	L STG/SMG (TPJ)	6b, 14, 22, 27b
7	832	0.015	4.30	−54	2	24	L PreCG (6v3)	6b, 8, 21, 22
8	696	0.018	4.85	62	−8	0	R STG (Te 2.1)	6b, 14, 22
9	696	0.016	4.39	48	8	2	R frontal operculum (Op6)	6b, 8, 16, 21
10	688	0.016	4.55	46	−22	8	R Heschl (Te 1.1)	6b, 27b
		0.011	3.53	50	−30	4	R STS (STS1)	
11	656	0.014	3.97	−4	10	40	L CG, anterior division*	8, 16, 20, 22
12	648	0.014	3.96	50	−10	40	R PostCG (3b)	6b, 22

Peak coordinates are reported in MNI space. Cytoarchitectonic labels were assigned using the Julich-Brain probabilistic atlas (v3.1)

*L* Left, *R* Right, *STS* Superior temporal sulcus, *PostCG* Postcentral gyrus, *IPL* Inferior parietal lobule, *STG* Superior temporal gyrus, *preSMA* Pre-supplementary motor area, *TPJ* Temporo-parietal junction, *SMG* Supramarginal gyrus, *PreCG* Precentral gyrus, *CG* cingulate gyrus

*For peak coordinates falling within cytoarchitectonic transition zones (GapMaps), or for which no probabilistic assignment to a specific cortical area was available, anatomical labeling was performed using the macroscopic Harvard-Oxford cortical atlas or the Harvard-Oxford subcortical atlas, both accessed via FSLeyes

###  Contrast analyses between word and pseudoword repetition

**Fig. 3:**
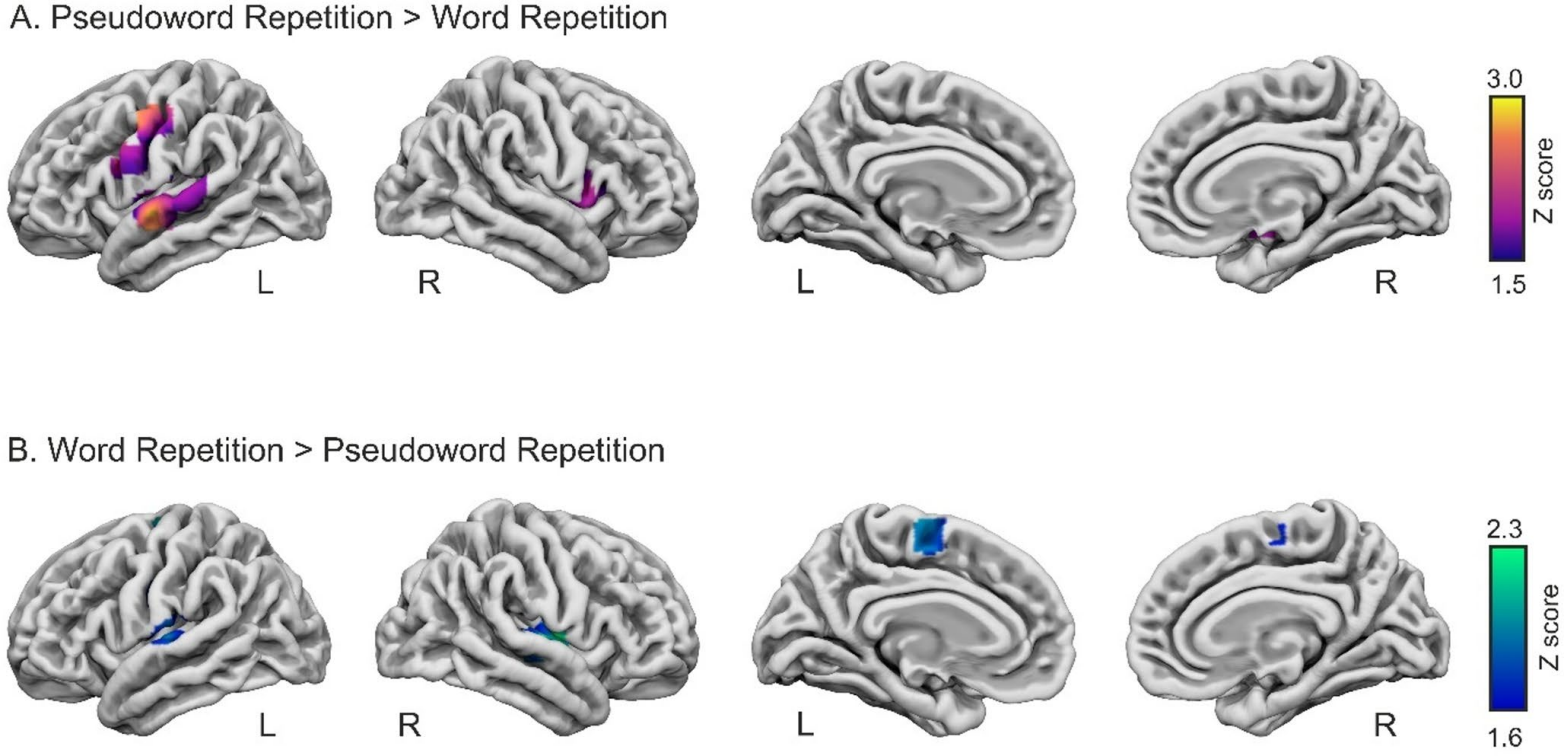
Results of the ALE meta-analysis subtraction contrast between pseudoword and word repetition: **A** Pseudoword Repetition > Word Repetition and **B** Word Repetition > Pseudoword Repetition. *L* Left, *R* Right

The contrast pseudoword repetition > word repetition yielded six significant clusters (Fig. 3A; Table 5 and Table S4). Cluster 1 was located in the left posterior STG and involved auditory associative cortex (Te3). Cluster 2 was subcortical and involved the right pallidum and putamen, with an additional local maximum in the amygdala; however, cluster composition analysis as provided by GingerALE indicated that the cluster extent was confined to the lentiform nucleus (see Table S4). Cluster 3 showed a peak in the left PreCG (6v3), whereas cluster composition analysis indicated predominant somatosensory involvement (see Table S4). For cluster 4, most of the cluster extent was located within the IFG, despite the peak being located at the PreCG. Cluster 5 was located in the left STG, corresponding to auditory associative cortex (Te 2.2). Finally, cluster 6 was located in the right frontal cortex, involving rostral premotor cortex within the PreCG (6r1) with a peak in the right IFG (44).

The reverse contrast, word repetition > pseudoword repetition, revealed four significant clusters (Fig. 3B; Table 5 and Table S4). Cluster 1 was located in the right temporal cortex and involved primary auditory cortex in Heschl’s gyrus (Te 1.0), with extension into auditory associative regions of the STG (Te 2.1). Cluster 2 was located in the left temporal cortex and similarly involved auditory associative cortex (Te 2.1) with additional involvement of primary auditory cortex (Te 1.0). Cluster 3 was centered in the left medial frontal cortex and involved dorsal premotor cortex in the PreCG (6d1), with extension into the SMA (6mp). Cluster 4 was located in the left sensorimotor region and involved primary somatosensory cortex in the PostCG (3a).

**Table 5:** Results for subtraction analyses

Contrast	Cluster	Size (mm^3^)	P	Z peak	x	y	Z	Peak Label
Pseudoword repetition > Word repetition	1	904	0.001	3.06	−62	−8	−6	L STG (Te3)
			0.003	2.78	−64	−12	−8	L STG (Te3)
	2	560	0.025	1.95	22	−4	−4	R pallidum*
			0.025	1.95	24	−4	−8	R pallidum*
			0.028	1.91	24	4	−6	R putamen*
			0.029	1.90	26	−4	−4	R putamen*
			0.029	1.90	26	−4	−12	R Amygdala*
			0.030	1.88	24	−4	0	R pallidum*
	3	472	0.003	2.75	−54	−6	42	L PreCG (6v3)
	4	400	0.008	2.42	−50	4	26	L PreCG (6v3)
	5	240	0.022	2.02	−66	−28	10	L STG (Te 2.2)
	6	200	0.014	2.21	50	10	8	R PreCG (6r1)
			0.004	1.80	46	12	12	R IFG (44)
Word repetition > Pseudoword repetition	1	792	0.004	2.69	50	−10	4	R Heschl (Te 1.0)
			0.004	2.68	52	−14	2	R STG (Te 2.1)
	2	440	0.015	2.17	−56	−18	10	L STG (Te 2.1)
			0.022	2.01	−52	−14	6	L Heschl (Te 1.0)
	3	376	0.019	2.07	−10	−8	64	L PreCG (6d1)
			0.025	1.96	−6	−10	64	L SMA (6mp)
	4	224	0.026	1.94	−46	−10	28	L PostCG (3a)
			0.030	1.88	−44	−16	28	White matter

Peak coordinates are reported in MNI space. Cytoarchitectonic labels were assigned using the Julich-Brain probabilistic atlas (v3.1)

*L* Left, *R* Right, *STG* Superior temporal gyrus, *PreCG* Precentral gyrus, *IFG* Inferior frontal gyrus, *SMA* Supplementary motor area, *PostCG* Postcentral gyrus

*For peak coordinates falling within cytoarchitectonic transition zones (GapMaps), or for which no probabilistic assignment to a specific cortical area was available, anatomical labeling was performed using the macroscopic Harvard-Oxford cortical atlas or the Harvard-Oxford subcortical atlas, both accessed via FSLeyes

###  Jackknife sensitivity analysis results

Only clusters that remained significant across all jackknife iterations were considered robust and are therefore emphasized in the discussion. Table 6 summarizes the original clusters that showed full reproducibility across all jackknife iterations, while Table S5 lists clusters that failed to survive all sensitivity tests. For ALE 1, five out of seven identified clusters remained significant across all replications, indicating high robustness. For ALE 2, clusters 1 to 5 were consistently reproduced across all jackknife analyses, whereas smaller clusters (6–9) failed to survive all iterations. For ALE 3, four clusters showed full robustness, while the remaining clusters exhibited limited reproducibility across jackknife analyses.

### Lateralization indices results

LIs computed from thresholded ALE-score images revealed a clear left-hemispheric dominance for general repetition (LI of 0.42) and pseudoword repetition (LI of 0.44). Word repetition showed a weaker, yet still left-lateralized, pattern (LI of 0.26). Together, these results indicate a predominant involvement of the left hemisphere in verbal repetition, with subtle differences in the degree of lateralization across stimulus types.

**Table 6:** Clusters that survived jack-knife analysis

ALE	Cluster	Size (mm3)	ALE peak	Z peak		x	y	z	Peak Label
ALE 1	1	15120	0.013- 0.039		3.21- 7.01	−48/−56/ −58/−54	−12/−18/−26/ −30/−4/−6/−42	35/6/8/22/10/26	L PostCG/PreCG/STG
	2	6536	0.018- 0.032		4.03- 6.05	48/62	−24/−32/−8/ −28	8/0/2	R STG
	3	3232	0.045		7.77	−6	−4	58	L SMA/preSMA
	4	2712	0.018- 0.029		5.45- 5.69	52/48/54	−8/−10/−4	26/38/42	R PostCG/PreCG
	5	1464	0.024		5.02	−6	10	38	L CG (ant. division)
ALE 2	1	6024	0.015- 0.034		3.96- 6.79	−56/−52/−40	−18/−26/−4/ −32	6/2/12	L STG/Heschl
	2	5816	0.012- 0.028		3.42- 6.06	50/52/58/ 46	−24/−18/−6/−8	6/2/4/8	R STG/POperc
	3	3792	0.021- 0.028		4.87- 5.95	−46/−58	−12/−4	34/22	L PostCG/PreCG
	4	2632	0.021- 0.034		4.88- 6.91	−6/4	−4/−2	60/62	L SMA/preSMA
	5	1904	0.023- 0.025		5.29- 5.56	54/48	−8/−10	26/38	R PostCG
ALE 3	2	1696	0.023		5.74	−60	−12	−2	L STS
	3	1360	0.020		5.21	−52	−12	42	L PostCG
	5	1296	0.018–0.019.018.019		4.89- 4.93	−4	0/−2	60/56	L preSMA
	6	1112	0.021		5.39	−62	−28	6	L STG/SMG (TPJ)

Peak labels reflect the spatial extent of cluster maxima across iterations rather than a single focal coordinate

*PostCG* postcentral gyrus, *PreCG* precentral gyrus, *STG* superior temporal gyrus, *SMA* supplementary motor area, *preSMA* pre-supplementary motor area, *CG* cingulate gyrus, *POperc* parietal operculum, *STS* superior temporal sulcus, *SMG* supramarginal gyrus, *TPJ* temporo-parietal junction

## Discussion

In the current study, we used ALE to conduct a coordinate-based meta-analysis of neuroimaging experiments investigating the neural basis of verbal repetition in healthy participants. The meta-analysis pooled 27 experiments employing in-scanner tasks that required the repetition of verbal material, mainly words and pseudowords. The analyses identified a set of brain regions that were consistently engaged across studies during verbal repetition tasks, as confirmed by jackknife sensitivity tests. These regions included auditory-phonological areas within the STG, encompassing primary and associative auditory cortices, motor planning and execution regions (PreCG, PostCG, SMA, preSMA), as well as regions associated with performance monitoring and cognitive control, including the ACC. The core auditory-motor network identified here largely overlaps with regions traditionally associated with the dorsal speech processing stream (Hickok and Poeppel 2007) and is thought to support auditory-motor integration mechanisms that are essential for speech production, verbal working memory, and language learning (López-Barroso et al. 2013, 2015; Assaneo et al. 2019; Orpella et al. 2022).

Taken together, these results suggest that verbal repetition is best understood as a composite function that bridges receptive and expressive language processes. Rather than reflecting isolated perceptual or motor mechanisms, repetition relies on the coordinated activity of auditory, phonological, articulatory, and control-related networks operating over transient time scales. Lateralization analyses further indicated a predominant contribution of the left hemisphere, with a stronger leftward bias for pseudoword repetition than for word repetition. Importantly, this left-hemispheric dominance should be interpreted as quantitative rather than absolute, as the ALE maps revealed robust bilateral engagement of homologous regions in both hemispheres.

The following sections examine the contributions of these subsystems in greater detail, situate the present findings within prior neuroimaging and lesion literature, and consider their theoretical and clinical implications. In doing so, they draw on converging evidence from neuroimaging and lesion studies and relate the results to contemporary models of verbal working memory, phonological learning, and language acquisition. In the discussion of the main ALE analyses (ALE 1 to 3), emphasis is placed on clusters that proved robust across all jackknife sensitivity tests.

###  Auditory and phonological processing

In the general repetition analysis (ALE 1), which pooled word and pseudoword repetition experiments, robust convergence was observed in bilateral auditory associative regions of the STG, with additional involvement of the right superior temporal sulcus. These regions constitute core auditory-phonological processing areas and have been consistently implicated in higher-level auditory and phonological aspects of speech processing beyond primary acoustic processing (Price 2012; Ozker et al. 2018; Yi et al. 2019; Ramos Nuñez et al. 2020). Their consistent engagement in ALE 1 suggests that verbal repetition, irrespective of stimulus type, relies on detailed auditory-phonological analysis supporting the transformation of incoming speech signals into articulatory representations.

When repetition conditions were examined separately, the core temporal pattern identified in ALE 1 was differentially extended depending on stimulus type. In ALE 2 (word repetition), convergence within the STG was accompanied by additional involvement of the primary auditory cortex in Heschl’s gyrus (Te 1.1 and Te 1.2), bilaterally, consistent with greater reliance on early auditory representations when repeating familiar, well-specified speech forms (Scott and Johnsrude 2003; Hickok and Poeppel 2007; Price 2012). By contrast, ALE 3 (pseudoword repetition) revealed robust convergence in the left superior temporal sulcus (STS1) and in the left temporo-parietal junction (STG/supramarginal gyrus interface). The superior temporal sulcus has been associated with higher-order auditory and phonological integration, particularly under increased perceptual or articulatory demands, and with the processing of unfamiliar or acoustically complex speech material (Hickok and Poeppel 2007; Rauschecker and Scott 2009; Raettig and Kotz 2008).

Converging evidence from lesion and clinical neuroimaging studies further characterizes this functional role, indicating that lesions involving the left posterior STG and temporo-parietal regions are associated with deficits in single-word comprehension (Hillis et al. 2017), phonological processing (Robson et al. 2012), and sentence repetition (Selnes et al. 1985), whereas right STG lesions affect syllable discrimination, supporting the notion of a bilaterally organized phonological processing system (Rogalsky et al. 2022). Consistent with these findings, neuroimaging evidence in post-stroke mixed transcortical aphasia has shown that preserved repetition performance is associated with bilateral activation of the mid-to-posterior portions of both STG and the middle temporal gyrus (López-Barroso et al. 2023).

At a systems level, this pattern can be interpreted within the framework of dorsal auditory-motor integration, in which superior temporal and temporo-parietal regions support phonological working memory by enabling the temporary buffering and maintenance of phonological representations and their transformation into articulatory codes (Hickok and Poeppel 2007; Buchsbaum et al. 2011; Saur et al. 2008). Accordingly, their engagement during pseudoword repetition likely reflects increased demands on sublexical phonological buffering and sensorimotor integration when lexical support is reduced. Within this systems-level account, these mechanisms not only contribute to repetition but also support sublexical stages of phonological word learning in adults, by enabling the temporary maintenance and rehearsal of unfamiliar sound sequences (Hickok and Poeppel 2007; López-Barroso et al. 2013, 2015; Lee et al. 2007) and are similarly engaged during phonological development in children (Booth et al. 2004), as proposed by theoretical accounts emphasizing the role of dorsal auditory-motor pathways in language acquisition and learning (Dick et al. 2014).

Taken together, these findings suggest that auditory associative regions of the STG form the core temporal substrate for verbal repetition across stimulus types.

###  Speech motor planning and speech articulation

Across analyses, robust convergence was observed in motor-related regions, with a differentiated pattern across repetition conditions. In ALE 1 (general repetition) and ALE 2 (word repetition), jackknife-stable convergence in the PreCG encompassed primary motor cortex **(**area 4b in ALE 1 and areas 4p/4a in ALE 2), together with primary somatosensory cortex in the PostCG (area 3b), bilaterally. This pattern is consistent with the established involvement of primary motor cortex in articulatory execution and of PostCG in processing somatosensory feedback related to articulatory movements, which is critical for accurate articulation (Gajardo-Vidal et al. 2021; Silva et al. 2022; Wilson et al. 2022). Auditory feedback mechanisms support feedback-based error detection and correction of articulatory gestures during speech production (Houde and Chang 2015), and therefore during repetition. Evidence from patients with acquired motor speech and language disorders provides direct support for this functional interpretation, as lesions involving the PreCG often cause apraxia of speech (Basilakos et al. 2015; Itabashi et al. 2016).

In contrast, ALE 3 (pseudoword repetition) did not show jackknife-stable convergence in primary motor cortex. Instead, consistent involvement was observed in primary somatosensory cortex (area 3b) and in medial frontal motor regions, specifically the preSMA. This pattern may reflect a relative shift away from primary motor execution toward higher-level motor planning and control processes during pseudoword repetition. Across repetition conditions, convergent engagement of somatosensory (area 3b) and medial frontal motor regions is consistent with their contribution to articulatory rehearsal mechanisms supporting verbal working memory (Baddeley 2003; Buchsbaum and D’Esposito 2008; Acheson and MacDonald 2009).

In the medial frontal cortex, convergence across ALE 1 and ALE 2 encompassed both the SMA and the preSMA, whereas in ALE 3 peak convergence was restricted to the preSMA. This dissociation aligns with previous accounts linking the SMA to the initiation and execution of well-learned motor sequences, and the preSMA to higher-level motor planning and sequencing under increased task demands (Tanji 1994; Ziegler et al. 1997; Hertrich et al. 2016; Cona and Semenza 2017). Accordingly, SMA involvement in ALE 1 and ALE 2 is consistent with the production of familiar speech patterns, whereas selective preSMA involvement in ALE 3 may reflect increased demands on planning and sequencing novel articulatory patterns in the absence of pre-established motor routines.

Taken together, these findings indicate consistent involvement of motor-related regions, including primary motor, somatosensory, and medial frontal cortices, in supporting speech production processes during verbal repetition.

###  Higher level language processing and domain general control

Beyond their role in motor planning and execution, medial frontal regions, particularly the preSMA, have been consistently implicated in higher-level control processes during speech and language production (Hertrich et al. 2016; Cona and Semenza 2017). In the present meta-analysis, robust convergence in medial frontal regions was observed across all three ALE analyses, with reliable peaks in the SMA/preSMA for ALE 1 and ALE 2, and in the preSMA for ALE 3. This pattern suggests a graded involvement of medial frontal control mechanisms depending on task demands. The SMA-preSMA complex is thought to exert supervisory control over action selection, sequencing, and initiation (Hartwigsen et al. 2013; Hope et al. 2014), potentially mediated through interactions with dorsal premotor regions (Hartwigsen et al. 2013) to regulate complex movement sequences during speech (Alario et al. 2006). While the SMA proper is more closely linked to the initiation and execution of well-learned motor routines, the preSMA appears particularly relevant for situations requiring increased cognitive control, flexible sequencing, and selection among competing motor plans, especially when speech production relies on non-routinized articulatory sequences as in pseudoword repetition.

In this context, it is noteworthy that ACC involvement was additionally observed primarily in ALE 1, the most inclusive analysis including all repetition conditions and the largest number of experiments and appeared again in ALE 3 but did not survive jackknife sensitivity analyses. This pattern suggests that ACC recruitment is not a robust feature of repetition per se, but varies with task context. Consistent with its established role in conflict monitoring and cognitive control (Carter et al. 1998; Botvinick et al. 2004; Abutalebi et al. 2012), ACC engagement during repetition may reflect supervisory monitoring processes recruited under increased phonological competition, such as during the processing of unfamiliar or non-semantic material (Hofmann et al. 2008).

Taken together, these findings suggest that verbal repetition engages not only sensorimotor execution systems but also a medial frontal control network centered on the SMA-preSMA complex, with ACC involvement that varies depending on task demands.

###  Differences between word and pseudoword repetition

Subtraction analyses comparing word and pseudoword repetition revealed systematic differences related to stimulus type. Importantly, although largely overlapping systems were engaged by both tasks, as shown by the individual ALE analyses, differences emerged at the level of specific cytoarchitectonic subregions. In particular, both contrasts involved regions within the STG and PreCG, but in distinct subregions, indicating modulation within shared auditory-motor networks rather than the recruitment of entirely separate systems.

Within the STG, both contrasts implicated associative auditory cortex, but in distinct subregions along the anterior-posterior axis. Likewise, within the PreCG, differential involvement of premotor versus more dorsal or medial motor-related subdivisions suggests that stimulus familiarity modulates the balance between articulatory planning and execution-related processes, rather than overall motor engagement.

In the pseudoword > word contrast, greater convergence of activation was observed in the left associative STG, including anterior and middle subdivisions (Te 3 and Te 2.2). These regions are implicated in phonological analysis and the processing of unfamiliar sound patterns, and have been shown to respond more strongly to pseudowords than words, particularly within associative auditory cortex (Newman and Twieg 2001; Majerus et al. 2005). Beyond this familiarity effect, increased recruitment of associative STG during pseudoword repetition may reflect heightened demands on auditory monitoring processes, as internal predictions are less precise in the absence of established lexical representations (Hashimoto and Sakai 2003; Tourville et al. 2008; Hickok et al. 2011).

Within the PreCG, pseudoword repetition showed greater convergence in premotor subdivisions, specifically left ventral premotor cortex (6v3), together with right premotor cortex (6r1) and right IFG (pars opercularis). These regions are associated with articulatory planning, sensorimotor integration, and the assembly of novel speech motor sequences. Consistent with this interpretation, prior fMRI studies have reported increased recruitment of premotor and posterior inferior frontal regions during the processing of unfamiliar or low-frequency phonological sequences, reflecting increased demands on articulatory code assembly (Papoutsi et al. 2009; Hope et al. 2014). Converging lesion evidence further indicates that damage to frontal-premotor regions and associated white-matter pathways is associated with impairments in phonological and motor aspects of speech production (Gajardo-Vidal et al. 2021; Wilson et al. 2022). At a systems level, stimulus-dependent differences are consistent with modulation within the dorsal auditory-motor pathway linking associative superior temporal regions with frontal premotor areas (Saur et al. 2008). This network is thought to be particularly engaged when speech production relies on the assembly and mapping of unfamiliar phonological sequences onto articulatory plans, as in pseudoword repetition and early stages of new word learning (López-Barroso et al. 2013, 2015).

Subcortical convergence differences were also observed: pseudoword repetition showed greater convergence in the right basal ganglia (globus pallidus and putamen) and in the right amygdala. These structures have been implicated in motor sequencing and control processes relevant to sublexical speech production (Vigneau et al. 2011; Oberhuber et al. 2013), as well as in the inhibition of competing motor programs (Mink 1996; Peeva et al. 2011; Beukema et al. 2015). This suggests that pseudoword repetition engages additional control resources to inhibit familiar syllabic patterns and generate novel articulatory sequences.

Conversely, word > pseudoword revealed greater convergence in the right primary auditory cortex (Te1.0; Heschl’s gyrus) and bilateral posterior associative STG (Te2.1), indicating more consistent recruitment of early and posterior auditory-phonological regions during word repetition. This pattern likely reflects more efficient and stereotyped auditory-lexical mapping for familiar stimuli (MacGregor et al. 2012). Importantly, this finding does not contradict the greater involvement of more anterior associative STG regions observed for pseudoword repetition. Rather, both contrasts point to a functional dissociation within auditory cortex, whereby word repetition preferentially engages posterior and primary auditory regions supporting efficient phonological-lexical mapping, while pseudowords recruit more anterior and middle associative STG regions associated with increased phonological analysis and monitoring demands.

Beyond auditory cortex, greater convergence for word was also observed in left medial and sensorimotor regions, including SMA (6mp), dorsal premotor cortex (PreCG; 6d1), and PostCG (3a). These differences may reflect memory retrieval of well-established articulatory motor programs associated with familiar words. Prior work has linked the SMA to the retrieval and initiation of overlearned speech motor sequences (Tremblay and Small 2011), while PreCG and PostCG support efficient articulatory execution and somatosensory monitoring once motor routines are automatized (Houde and Chang 2015; Gajardo-Vidal et al. 2021; Wilson et al. 2022). Together, these findings suggest that word repetition preferentially engages streamlined auditory-motor pathways that capitalize on stored lexical and motor representations, in contrast to pseudoword repetition, which relies more heavily on effortful sublexical assembly and control mechanisms. Overall, these contrast analyses should be regarded as exploratory and interpreted with caution, particularly in light of methodological variability across studies, including differences in task execution mode (overt vs. covert repetition).

###  Clinical implications

The present findings may provide a useful synthesis for clinical research on repetition and neurorehabilitation. By delineating a core dorsal auditory-motor network consistently engaged across repetition tasks, this meta-analysis provides a neuroanatomical framework for interpreting repetition impairments commonly observed in acquired language disorders. The stable involvement of superior temporal, premotor, and medial frontal regions converge with rehabilitation approaches that exploit auditory-motor coupling and repetition-based practice, such as Imitation-Based Aphasia Therapy (Duncan and Small 2016) and Speech Entrainment Therapy (Fridriksson et al. 2012). Moreover, the engagement of regions associated with verbal working memory aligns with the clinical use of repetition-based tasks to assess and train verbal short-term memory in aphasia, including assessment batteries such as the Temple Assessment of Language and Short-Term Memory in Aphasia (TALSA; Martin et al. 2018). At a translational level, identifying brain regions that are reliably involved in repetition may help guide hypothesis-driven research on neuromodulation targets. These implications should be regarded as contextual and provisional, serving to situate the present findings within existing clinical practices rather than to prescribe specific therapeutic interventions.

###  Limitations

First, several limitations are intrinsic to the ALE approach. ALE relies on peak activation coordinates reported in published studies rather than full statistical maps, limiting sensitivity to effect sizes, subthreshold activations, and fine-grained spatial variability. In addition, although ALE results are defined at the cluster level, anatomical interpretation is based on cytoarchitectonic labeling of cluster peaks. This approach captures the dominant anatomical contributors to each cluster but may not fully reflect the spatial extent of clusters, which may encompass adjacent regions not expressed as local maxima. Methodological heterogeneity across studies, including differences in baseline conditions and control tasks, is also inherent to coordinate-based meta-analyses and may influence the degree of convergence observed across experiments.

Second, additional limitations are related to characteristics of the available dataset. The inclusion of both left- and right-handed participants may introduce variability related to language lateralization. However, substantial overlap in language dominance across handedness groups (Knecht et al. 2000a, b) suggests excluding left-handed participants was not warranted. In addition, it is worth mentioning that subgroup analyses focusing on pseudoword repetition (ALE 3) should be interpreted with caution due to the relatively small number of experiments available (*n* = 10). Moreover, task execution mode was not fully balanced across conditions, as all word repetition studies involved overt responses whereas a subset of pseudoword studies employed covert repetition. Although jackknife analyses indicated that the main ALE clusters were not driven by any single experiment, residual effects of execution mode cannot be entirely excluded. Finally, the scope of the present synthesis was constrained by the limited availability of sentence repetition experiments, highlighting the need for future work across different linguistic units and levels of complexity.

## Conclusions

This meta-analysis identified a robust, bilaterally distributed dorsal network supporting verbal repetition, encompassing auditory-phonological regions of the superior temporal cortex, sensorimotor cortices (PreCG, PostCG), and medial frontal motor regions (preSMA/SMA), with limited and non-core involvement of the ACC. Lateralization analyses revealed a predominant left-hemispheric contribution across conditions, with a stronger leftward bias for pseudoword than for word repetition, despite the largely bilateral engagement observed in ALE patterns. Direct contrasts further showed that pseudoword repetition preferentially engaged left posterior auditory associative cortex, ventral premotor regions at the PreCG-IFG interface, and basal ganglia, whereas word repetition showed stronger involvement of primary auditory cortex and medial frontal motor regions. Overall, the present findings do not reflect a strict ventral-dorsal dissociation between word and pseudoword repetition, but rather graded shifts in the relative engagement of specific subregions within a shared dorsal auditory-motor architecture, consistent with flexible reweighting and reliance on sublexical phonological processing, auditory-motor mapping, and articulatory implementation as a function of stimuli familiarity.

## Supplementary Information

Below is the link to the electronic supplementary material.

Supplementary Material 1

## Data Availability

The MNI coordinates used for the three ALE meta-analyses and the statistical maps derived from the Ginger ALE analysis are available at OSF repository (https://osf.io/w6s87/).
