# Supplementary material for: Mapping the neural patterns of verbal repetition: an activation likelihood estimation meta-analysis
Source: Brain Struct Funct. 2026 May 30;231(5):75. doi: 10.1007/s00429-026-03119-3 (PMC13221332; doi:10.1007/s00429-026-03119-3)
Supplement: Supplementary file 1 — Supplementary Material 1 [file 429_2026_3119_MOESM1_ESM.pdf]

Supplementary Material

Mapping the Neural Patterns of Verbal Repetition: An Activation Likelihood Estimation Meta-Analysis

Ariane Awana, Marcelo L. Berthier, María José Torres-Prioris* and Diana López-Barroso*

*These authors contributed equally to this work

Corresponding authors:

Diana López-Barroso. E-mail address: dlopbarroso@uma.es

María José Torres-Prioris. E-mail address: mjprioris@uma.es

Table S1. Anatomical composition of significant clusters for ALE 1 (general repetition).

Cluster	Hemisphere (%)	Lobe composition (%)	Anatomical subregions (%)	Cytoarchitectonic regions/nuclei (%)
1	Left 100%	Temporal 66.2%; Frontal 27.5%; Parietal 6.3%	Superior Temporal Gyrus 47.8%; Precentral Gyrus 24.2%; Transverse Temporal Gyrus 10.2%; Middle Temporal Gyrus 8.1%; Postcentral Gyrus 6.8%; Inferior Frontal Gyrus 3.0%	BA22 30.8%; BA41 23.8%; BA6 14.7%; BA4 7.3%; BA21 4.5%; BA3 4.3%; BA43 4.1%; BA13 1.8%; BA9 1.8%; BA42 1.4%; BA44 1.3%
2	Right 100%	Temporal 72.9%; Sub-lobar 21.8%; Frontal 5.2%	Superior Temporal Gyrus 56.0%; Insula 20.9%; Transverse Temporal Gyrus 15.7%; Precentral Gyrus 5.2%; Middle Temporal Gyrus 2.2%	BA41 32.3%; BA22 31.4%; BA13 27.7%; BA6 3.1%; BA21 2.2%; BA43 1.8%
3	Left 84.9%; Right 15.1%	Frontal 100%	Medial Frontal Gyrus 96.6%; Paracentral Lobule 1.7%; Superior Frontal Gyrus 1.7%	BA6 98.3%; BA31 1.7%
4	Right 100%	Frontal 100%	Precentral Gyrus 100%	BA6 60.9%; BA4 39.1%
5	Left 98.9%; Right 1.1%	Limbic 91.6%; Frontal 8.4%	Cingulate Gyrus 91.6%; Medial Frontal Gyrus 8.4%	BA24 63.2%; BA32 36.8%
6	Right 100%	Sub-lobar 71.4%; Frontal 28.6%	Insula 71.4%; Precentral Gyrus 28.6%	BA13 66.2%; BA44 28.6%
7	Left 100%	Sub-lobar 100%	Thalamus 99.2%	Medial Dorsal Nucleus 26.4%; Ventral Lateral Nucleus 17.4%; Mammillary Body 14.9%; Ventral Posterior Lateral Nucleus 10.7%; Ventral Posterior Medial Nucleus 5.0%; Pulvinar 1.7%

Note. Anatomical labeling and relative subregional contributions for each significant ALE cluster derived from GingerALE cluster-level Talairach atlas output (gray matter only).

Table S2. Anatomical composition of significant clusters for ALE 2 (word repetition).

Cluster	Hemisphere (%)	Lobe composition (%)	Anatomical subregions (%)	Cytoarchitectonic regions/nuclei (%)
1	Left 100%	Temporal 86.9%; Frontal 11.3%; Sub-lobar 1.8%	Superior Temporal Gyrus 60.4%; Transverse Temporal Gyrus 18.2%; Precentral Gyrus 12.0%; Middle Temporal Gyrus 8.0%; Insula 1.5%	BA22 43.6%; BA41 35.6%; BA6 8.4%; BA13 4.0%; BA43 3.3%; BA21 1.5%
2	Right 100%	Temporal 61.5%; Sub-lobar 30.9%; Frontal 7.6%	Superior Temporal Gyrus 45.2%; Insula 30.2%; Transverse Temporal Gyrus 16.6%; Precentral Gyrus 7.6%	BA13 36.5%; BA41 33.9%; BA22 20.6%; BA6 5.0%; BA43 2.0%
3	Left 100%	Frontal 78.8%; Parietal 21.2%	Precentral Gyrus 76.9%; Postcentral Gyrus 23.1%	BA6 44.2%; BA4 36.5%; BA43 11.5%; BA3 7.7%
4	Left 83.1%; Right 16.9%	Frontal 100%	Medial Frontal Gyrus 100%	BA6 100%
5	Right 100%	Frontal 100%	Precentral Gyrus 100%	BA6 88.9%; BA4 11.1%
6	Right 100%	Frontal 74.1%; Parietal 25.9%	Postcentral Gyrus 55.6%; Precentral Gyrus 27.8%; Paracentral Lobule 16.7%	BA4 72.2%; BA3 27.8%
7	Left Cerebellum 100%	Anterior Lobe 75.7%; Posterior Lobe 24.3%	Culmen 44.7%; Declive 21.4%; Fastigium 1.9%	Dentate 32.0%
8	Right Cerebellum 100%	Posterior Lobe 51.0%; Anterior Lobe 49.0%	Declive 51.0%; Culmen 37.8%; Fastigium 1.0%	Dentate 10.2%
9	Right 100%	Sub-lobar 94.3%; Frontal 5.7%	Insula 94.3%; Precentral Gyrus 5.7%	BA13 82.9%; BA44 5.7%

Note. Anatomical labeling and relative subregional contributions for each significant ALE cluster derived from GingerALE cluster-level Talairach atlas output (gray matter only).

Table S3. Anatomical composition of significant clusters for ALE 3 (pseudoword repetition).

Cluster	Hemisphere (%)	Lobe composition (%)	Anatomical subregions (%)	Cytoarchitectonic regions/nuclei (%)
1	Right 100%	Sub-lobar 100%	Lentiform Nucleus 98.3%; Caudate 1.7%	Putamen 77.3%; Lateral Globus Pallidus 21.0%; Caudate Body 1.7%
2	Left 100%	Temporal 100%	Superior Temporal Gyrus 94.9%; Middle Temporal Gyrus 5.1%	BA22 65.4%; BA21 28.2%
3	Left 100%	Parietal 54.7%; Frontal 45.3%	Postcentral Gyrus 54.7%; Precentral Gyrus 45.3%	BA3 52.8%; BA4 41.5%; BA6 3.8%; BA1 1.9%
4	Left 100%	Temporal 100%	Superior Temporal Gyrus 62.2%; Transverse Temporal Gyrus 37.8%	BA41 95.6%; BA42 2.2%
5	Left 100%	Frontal 100%	Medial Frontal Gyrus 100%	BA6 100%
6	Left 100%	Temporal 100%	Superior Temporal Gyrus 51.0%; Middle Temporal Gyrus 49.0%	BA22 53.1%; BA42 14.3%; BA21 10.2%; BA41 8.2%
7	Left 100%	Frontal 100%	Precentral Gyrus 52.0%; Inferior Frontal Gyrus 48.0%	BA6 52.0%; BA9 36.0%; BA44 12.0%
8	Right 100%	Temporal 100%	Superior Temporal Gyrus 100%	BA22 90.9%
9	Right 100%	Sub-lobar 55.3%; Frontal 44.7%	Insula 55.3%; Precentral Gyrus 44.7%	BA13 52.6%; BA44 44.7%
10	Right 100%	Temporal 75.0%; Sub-lobar 25.0%	Superior Temporal Gyrus 45.0%; Transverse Temporal Gyrus 30.0%; Insula 25.0%	BA41 50.0%; BA13 50.0%
11	Left 100%	Limbic 89.8%; Frontal 10.2%	Cingulate Gyrus 89.8%; Medial Frontal Gyrus 10.2%	BA24 59.2%; BA32 40.8%
12	Right 100%	Frontal 100%	Precentral Gyrus 100%	BA4 87.5%; BA6 12.5%

Note. Anatomical labeling and relative subregional contributions for each significant ALE cluster derived from GingerALE cluster-level Talairach atlas output (gray matter only).

Table S4. Anatomical composition of significant clusters for subtraction analyses.

Cluster	Hemisphere (%)	Lobe composition (%)	Regional composition (%)	Cytoarchitectonic regions /nuclei (%)
**Pseudoword repetition > Word repetition**				
1	Left 100%	Temporal 100%	Superior Temporal Gyrus 93.3%; Middle Temporal Gyrus 6.7%	BA22 60.0%; BA21 31.7%
2	Right 100%	Sub-lobar 100%	Lentiform Nucleus 100%	Putamen 71.0%; Lateral Globus Pallidus 29.0%
3	Left 100%	Parietal 67.7%; Frontal 32.3%	Postcentral Gyrus 67.7%; Precentral Gyrus 32.3%	BA3 64.5%; BA4 32.3%; BA1 3.2%
4	Left 100%	Frontal 100%	Inferior Frontal Gyrus 74.1%; Precentral Gyrus 25.9%	BA9 66.7%; BA6 25.9%; BA44 7.4%
5	Left 100%	Temporal 100%	Superior Temporal Gyrus 80.0%; Middle Temporal Gyrus 20.0%	BA42 50.0%; BA22 30.0%; BA21 10.0%
6	Right 100%	Sub-lobar 80.0%; Frontal 20.0%	Insula 80.0%; Precentral Gyrus 20.0%	BA13 80.0%; BA44 20.0%
**Word repetition > Pseudoword repetition**				
1	Right 100%	Sub-lobar 53.8%; Temporal 40.4%; Frontal 5.8%	Insula 53.8%; Superior Temporal Gyrus 38.5%; Precentral Gyrus 5.8%; Transverse Temporal Gyrus 1.9%	BA13 53.8%; BA22 38.5%; BA6 3.8%; BA41 1.9%
2	Left 100%	Temporal 100%	Superior Temporal Gyrus 56.0%; Transverse Temporal Gyrus 44.0%	BA41 64.0%; BA22 32.0%; BA42 4.0%
3	Left 100%	Frontal 100%	Medial Frontal Gyrus 100%	BA6 100%
4	Not assigned*	Not assigned*	Not assigned*	Not assigned*

Note. Anatomical labeling and relative subregional contributions for each significant ALE cluster derived from GingerALE cluster-level Talairach atlas output (gray matter only). *Clusters for which GingerALE did not return anatomical labels are reported as ‘Not assigned’.

Table S5. Clusters that did not survive jack-knife analysis.

ALE	Cluster	x	y	z	Experiments*
ALE 1	6	48	6	0	6
	7	-12/-20	-18/-14	4/8	5, 6, 8, 17, 22
ALE 2	6	20/22	-28/-26	62/72	5, 6a, 9
	7	-14/-24	-62	-20/-22	5, 6a, 10, 19
	8	16	-62	-18	5, 6a, 9, 10
	9	44	8	0	1, 2, 5, 6a, 10
ALE 3	1	28/20	-4/8	-8/4	6b, 22
	4	-52/-44	-34/-32	14/12	6b, 8
	7	-54	2	24	8, 21, 22
	8	62	-8	0	6b, 14, 22
	9	48	8	2	6b, 8, 16, 21
	10	46/50	-22/-30	8/4	6b, 24
	11	-4	10	40	8, 16, 20
	12	50	-10	40	6b, 8, 22

Note. *Experiments with unproportionate influence on the cluster.
